# Meta-DHGNN: method for CRS-related cytokines analysis in CAR-T therapy based on meta-learning directed heterogeneous graph neural network

**DOI:** 10.1093/bib/bbae104

**Published:** 2024-03-27

**Authors:** Zhenyu Wei, Chengkui Zhao, Min Zhang, Jiayu Xu, Nan Xu, Shiwei Wu, Xiaohui Xin, Lei Yu, Weixing Feng

**Affiliations:** Intelligent Systems Science and Engineering College, Harbin Engineering University, Harbin 150001, China; Intelligent Systems Science and Engineering College, Harbin Engineering University, Harbin 150001, China; Shanghai Unicar-Therapy BioMedicine Technology Co., Ltd, Shanghai, China; Intelligent Systems Science and Engineering College, Harbin Engineering University, Harbin 150001, China; Intelligent Systems Science and Engineering College, Harbin Engineering University, Harbin 150001, China; Shanghai Unicar-Therapy BioMedicine Technology Co., Ltd, Shanghai, China; School of Chemical and Molecular Engineering, East China Normal University, Shanghai 200000, China; Intelligent Systems Science and Engineering College, Harbin Engineering University, Harbin 150001, China; Intelligent Systems Science and Engineering College, Harbin Engineering University, Harbin 150001, China; Shanghai Unicar-Therapy BioMedicine Technology Co., Ltd, Shanghai, China; School of Chemical and Molecular Engineering, East China Normal University, Shanghai 200000, China; Intelligent Systems Science and Engineering College, Harbin Engineering University, Harbin 150001, China

**Keywords:** CAR-T immunotherapy, CRS, meta-learning, graph neural network, IFN

## Abstract

Chimeric antigen receptor T-cell (CAR-T) immunotherapy, a novel approach for treating blood cancer, is associated with the production of cytokine release syndrome (CRS), which poses significant safety concerns for patients. Currently, there is limited knowledge regarding CRS-related cytokines and the intricate relationship between cytokines and cells. Therefore, it is imperative to explore a reliable and efficient computational method to identify cytokines associated with CRS. In this study, we propose Meta-DHGNN, a directed and heterogeneous graph neural network analysis method based on meta-learning. The proposed method integrates both directed and heterogeneous algorithms, while the meta-learning module effectively addresses the issue of limited data availability. This approach enables comprehensive analysis of the cytokine network and accurate prediction of CRS-related cytokines. Firstly, to tackle the challenge posed by small datasets, a pre-training phase is conducted using the meta-learning module. Consequently, the directed algorithm constructs an adjacency matrix that accurately captures potential relationships in a more realistic manner. Ultimately, the heterogeneous algorithm employs meta-photographs and multi-head attention mechanisms to enhance the realism and accuracy of predicting cytokine information associated with positive labels. Our experimental verification on the dataset demonstrates that Meta-DHGNN achieves favorable outcomes. Furthermore, based on the predicted results, we have explored the multifaceted formation mechanism of CRS in CAR-T therapy from various perspectives and identified several cytokines, such as IFNG (IFN-γ), IFNA1, IFNB1, IFNA13, IFNA2, IFNAR1, IFNAR2, IFNGR1 and IFNGR2 that have been relatively overlooked in previous studies but potentially play pivotal roles. The significance of Meta-DHGNN lies in its ability to analyze directed and heterogeneous networks in biology effectively while also facilitating CRS risk prediction in CAR-T therapy.

## INTRODUCTION

In biomedical research, network-based analysis has emerged as a valuable tool for accomplishing various critical tasks. Graph embedding, also known as network embedding or graph representation learning, is employed to analyze and interpret graph data. This approach involves learning a condensed feature representation of each node in the graph, which can be utilized in tasks like community detection, link prediction, node classification and clustering. The objective is to reduce the graph’s dimensionality while preserving its structural information, thus enabling the utilization of more efficient and effective machine learning models. Traditional embedding methods like Laplacian feature maps [1] and matrix factorization(MF) have demonstrating promising results [2].

When analyzing graphs, certain methods like a random walk, deepwalk [2] and node2vec [3] have proven effective for node classification, while struc2vec [4] is better suited for link prediction. However, these methods are primarily applied to undirected homogeneous graphs. In reality, complex, directed and heterogeneous graphs are more reflective of real-world scenarios. Consequently, recent research has been focused on characterizing information on heterogeneous graphs, resulting in significant advancements, especially in the application of deep neural networks with heterogeneous graph data [5]. Most of the existing graph neural network (GNN) models assumed that there was only one type of graph node, which was connected through one type of edge. The heterogeneous GNNs have been widely utilized in the graph data mining tasks for example, link prediction [6], node classification [7] and node clustering [8]. The heterogeneous graph attention network (HAN) introduced heterogeneous structures and semantic-level attentions to the graph attention networks [9].

However, real-world graphs do not always obey the homophily assumption but show an opposite property, i.e. heterophily that linked nodes have dissimilar features and different class labels. For instance, in molecular networks, protein structures are more likely composed of different types of amino acids that are linked together [10]. But these are only heterogeneous analyses, in fact, many of them are directional relationships and may be one-way rather than two-way relationships. Learning from digraph (directed graph) data to solve practical problems, such as traffic prediction [11, 12], knowledge discovery [13] and time-series problems [14, 15], has attracted increasing attention. Therefore, it is necessary to have an algorithm that can take into account both heterogeneous relationships and directed relationships. This research direction is becoming a hot research topic due to its huge potential.

The immune system heavily relies on cytokines for communication and regulation. The relationship between cytokines and cells is intricate, diverse and pivotal for comprehending the immune system’s functionality. Many of these links represent directed and heterogeneous relationships. However, existing GNN methods lack directional and heterogeneous algorithms for node analysis, thereby hindering the analysis of relationships between human cytokines or any substances with node classification properties in biological problems. For CAR-T therapy, a more accurate and comprehensive exploration of cytokines is imperative. In this study, a combination of directed [16] and heterogeneous networks [7] was employed to identify the key cytokines associated with cytokine release syndrome (CRS) in CAR-T therapy. We combine directed modules on the basis of heterogeneous networks, which can process-directed information. The meta-learning framework was also utilized, along with directed heterogeneous networks, to address the issue, considering the correlation between all data samples. The result was the successful creation of the semi-supervised classification model, Meta-DHGNN, a directed heterogeneous GNN model that can effectively deal tacking this problem. Seldom research focused on integrating the meta- and heterogenous-information in the algorithm to analyze biological problems, which are makes our research novel and significant.

## DATA AND METHODS

### Heterogeneous public datasets

Since there are no directed and heterogeneous public datasets, we initially tested the model with heterogeneous public datasets and then conducted a second test on our own directed heterogeneous cytokine datasets.

The algorithm’s efficacy and practicality in this study were initially validated through experimentation on two datasets, namely, IMDB and ACM. The ACM dataset comprises 3025 papers (P), 5835 authors (A) and 56 topics (S). The total number of features amounts to 1830, with the training set comprising 600 samples, the validation set consisting of 300 samples and the test set encompassing 2125 samples. The IMDB dataset encompasses a total of 4780 films, with involvement from 5841 actors and direction by 2269 directors. The total number of features amounts to 1312, with the training set comprising 300 samples, the validation set consisting of 300 samples and the test set encompassing 2687 samples. Table 1 presents the experimental data as well as the training set, validation set and test set utilized during the training process.

**Table 1:** Data set. The table presents the ACM and IMDB datasets along with their respective number of features, while also illustrating the distribution proportions of the training, validation and test sets within each dataset

Dataset	Feature	Train	Validation	Test
ACM	1830	600	300	2125
IMDB	1312	300	300	2687

### Cytokine interaction network construction

**Figure 1:**
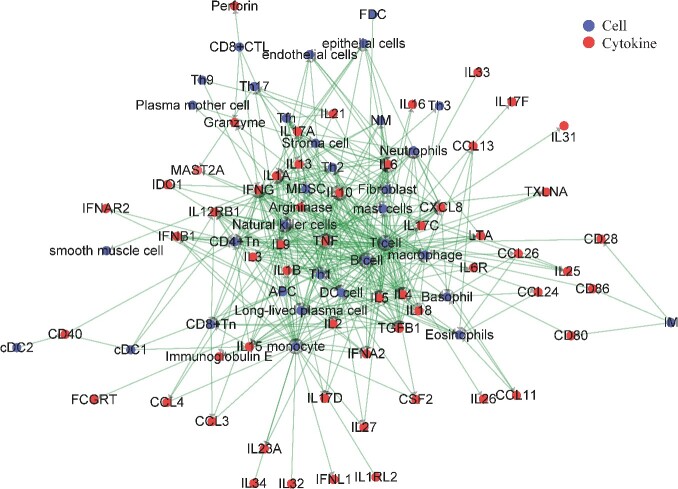
The core-directed heterogeneous topology diagram of cytokines and cells. This is what we did with Cytoscape software. It can be seen that the cell is basically concentrated in the middle part, and the cytokines (enzymes) are scattered on the side.

Firstly, a core-directed heterogeneous topology diagram of the relationship between cytokines and cells was constructed based on the extensive literature through our manually searching. The diagram, shown in Figure 1, comprises 57 cytokines (enzymes) and 33 cells, totaling 395 connections.

Among them, cytokines are released by cells, establishing an indirect connection between cytokines through cellular interactions. Cell-to-cell communication occurs via the release of cytokines or membrane proteins (receptors), and in this context, cell-to-cell connections are facilitated by cytokine signaling. If a cytokine is released by a cell, then the cell is directed to that cytokine, if a cell is affected by a cytokine, then the cytokine is directed to that cell. The influence exerted can either promote or inhibit cellular responses.

Aim to represent the nature of the connections between cytokines and cells, Figure 1 illustrates the directed connections between cytokines and cells as well as cell-to-cell interactions. The cells are depicted as blue nodes, while the cytokines are represented by red nodes.

About connections between cytokines, the keyword ‘Cytokine’ was used to search the NCBI website (https://www.ncbi.nlm.nih.gov/). This resulted in a list of 1769 cytokines, chemokines and soluble receptors. The STRING website (https://string-db.org/) was utilized to further understand how these cytokines interact, resulting in information on 1682 cytokines. These data were combined to create the final comprehensive directed heterogeneous network of cytokines and cells.

### Meta-DHGNN

This study presents a new approach to analyzing non-Euclidean graphical data that utilizes meta-learning, building on recent achievements in the field [17, 18]. Specifically, a semi-supervised classification model was developed called the directed heterogeneous GNN architecture based on meta learning (Meta-DHGNN).

The Meta-DHGNN model comprises a meta-learner and a base-learner that use the same GNN architecture. Initially, the meta-learner learns and optimizes the DHGNN model’s initial training parameters for better generalization ability. Afterward, the base learner trains the DHGNN model.

**Figure 2:**
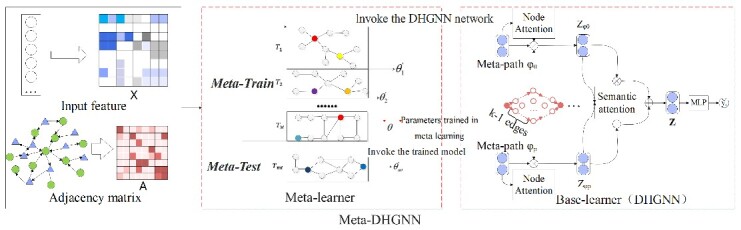
Meta-DHGNN model diagram. The leftmost section represents the input, adjacency matrix, and input features of the digital model. In the middle section lies the model’s meta-learning module, utilized for pre-training purposes. The far right of the model showcases the oriented heterogeneous module, with its central part representing the directed module and the remaining sections depicting node-level and semantic-level meta-paths within the heterogeneous module.

Like other GNN models, each DHGNN node collects information from its second-order neighborhood. The red arrow shows that the Adam method is used for gradient descent to optimize the model parameters. ${\theta}^{\prime}$ denotes the parameters after each meta-training, while $\theta$ denotes the parameters after all M meta-training. M denotes the number of meta-learning tasks. Figure 2 illustrates the main process. Specific steps were created to incorporate the directional and heterogeneous information of the network, which sets it apart from other algorithms.

In the third module, depicted as the Base-learner in Figure 2, the red component signifies the directed transformation within our model, corresponding to the adjacency matrix. Correspondingly, the blue element represents the integration of heterogeneous information through meta-paths in our model, initially consolidated via node-level attention and subsequently fused using semantic-level attention. Evidently, this amalgamation effectively combines directed information and heterogeneous information.

### Directed part

In this study, Markov chain attributes are utilized to solve the issue of directed graphs, specifically for graph G = (V, E) and its directed adjacency matrix A.

A Markov process with transition matrix ${P}_{rw}={D}^{-1}A$, where $D\left(\mathrm{I},\mathrm{i}\right)={\sum}_jA\left(i,j\right)$, is known as a random walk on G. Previous research has made a small adjustment to random walk algorithm and called it PageRank [19]. This modification involves adding a small probability of teleporting back to any node. The transition matrix used for PageRank is defined as ${P}_{pr}=\left(1-\alpha \right){P}_{rw}+\frac{\alpha }{n}{I}^{n\times n}$, where α∈(0, 1) represents the transmission probability [20]. With this property in mind, this article defines the digraph Laplacian using PageRank. ${\pi}_{pr}$ has analogy property with nodes degree matrix in undirected graph that reflecting the connectivity between nodes. This article refers to this as *L_pr_*, presented in symmetric normalized format [21] as follows:


(1) \begin{equation*} {L}_{pr}=I-\frac{1}{2}\left({\prod}_{pr}^{\frac{1}{2}}{P}_{pr}{\prod}_{pr}^{-\frac{1}{2}}+{\prod}_{pr}^{-\frac{1}{2}}{P}_{pr}^T{\prod}_{pr}^{-\frac{1}{2}}\right) \end{equation*}

In an undirected graph, one can replace the position of D with $\prod_{pr}=\frac{1}{{\left\Vert{\pi}_{pr}\right\Vert}_1} Diag\left({\pi}_{pr}\right)$. This study utilizes personalized PageRank and re-evaluates the PageRank equation ${P}_{pr}=\left(1-\alpha \right){P}_{rw}+\frac{\alpha }{n}{I}^{n\times n}$ to minimize computation. N indicates the number of rows. To create an auxiliary node plan, we introduce an auxiliary node ξ∈V as the personalized PageRank teleport set T = {ξ}. Using this approach, this article define the transition matrix of personalized PageRank *P*_ppr_ in the graph *G*_ppr_ as follows:


(2) \begin{equation*} {P}_{ppr}=\left[\begin{array}{l}\left(1-\alpha \right)\tilde{P}\\{}\frac{1}{n}{I}^{1\times n}\end{array}\right.\left.\begin{array}{l}\alpha{I}^{n\times 1}\\{}0\end{array}\right],{P}_{ppr}\in{R}^{\left(n+1\right)\times \left(n+1\right)} \end{equation*}

If letting $\tilde{\mathrm{P}}={\tilde{\mathrm{D}}}^{-1}\tilde{\mathrm{A}},\tilde{\mathrm{A}}=\mathrm{A}+{\mathrm{I}}^{\mathrm{n}\times \mathrm{n}}$ represent the adjacency matrix that includes a self-ring, ${P}_{ppr}$ is both aperiodic and irreducible. As a result, it has a unique left eigenvector, which can be denoted as ${\pi}_{ppr}\epsilon{R}^{n+1}$. However, it is worth noting that $\tilde{P\ }$ may not necessarily be irreducible. To simplify Equation 1, this article limit the range of α to a small value:


(3) \begin{align*} {L}_{\mathrm{appr}}=&I-\frac{1}{2}\left({\tilde{\varPi}}^{\frac{1}{2}}\tilde{P}{\tilde{\varPi}}^{-\frac{1}{2}}+{\tilde{\varPi}}^{-\frac{1}{2}}{\tilde{P}}^T{\tilde{\varPi}}^{\frac{1}{2}}\right)\nonumber\\&\approx I-\frac{1}{2}\left({\tilde{\varPi}}_{appr}^{\frac{1}{2}}\tilde{P}{\tilde{\varPi}}_{appr}^{-\frac{1}{2}}+{\tilde{\varPi}}_{appr}^{-\frac{1}{2}}{\tilde{P}}^T{\tilde{\varPi}}_{appr}^{\frac{1}{2}}\right) \end{align*}

As α approaches 1, our method becomes more similar to the undirected graph Laplacian. This means that α can determine the conversion level from a directed form to an undirected form. A smaller value of α preserves more directed properties, while a larger value does the opposite.

To achieve *k*-order proximity, this article define the *k*-order proximity matrix P^(k)^(k ∈ Z):


(4) \begin{equation*} {P}^{(k)}=\left\{\begin{array}{@{}l}I,k=0\\{}{\tilde{D}}^{-1}\tilde{A},k=1\\{} Inter\sec t\left({\left({P}^{(1)}\right)}^{K-1}{\left({P}^{(1)^T}\right)}^{K-1},{\left({P}^{(1)^T}\right)}^{K-1}{\left({P}^{(1)}\right)}^{K-1}\right)/2,k\ge 2\end{array}\right\} \end{equation*}

where $\tilde{A}$ is the adjacency matrix with self-loops of G, and $\tilde{D}$ is the corresponding diagonalized degree matrix. Intersect (.) denotes the element-wise intersection of matrices. The sum operation is only performed when the corresponding positions have meeting and diffusion paths, the sum operation is performed; otherwise, it is 0.

This study involves adding a residual connection and modifying the attention levels of multiple heads from 1 to 4. This makes the model more complex, but the residual connection can prevent the gradient disappearance problem. Additionally, the residual connection ensures that the improved model’s performance is enhanced compared to the original performance.

### Node attention

Because nodes can vary in their characteristics, each node type has a different feature space. A type-specific transformation matrix, ${M}_{\varnothing i}$, can be created for each type to project the features of various types of nodes into the same feature space. $\varnothing i$ is the type of each node. The projection process can be as follows:


(5) \begin{equation*} {\mathrm{h}}_i^{\prime}={M}_{\phi i}\cdot{h}_i \end{equation*}

where *h_i_* and *h*'*_i_* represent the original and projected features of node *i*, respectively.

The meta path refers to a sequence of nodes and edges within a complex network, which not only relies on the direct connections between nodes but also incorporates higher-order relationships among them. When looking at a particular meta path, the node-level attention ${e}_{ij}^{\varnothing }$ can learn the node j’s significance for node i. This significance can be expressed as the importance of the meta-path dependent node pair (*i*, *j*), which can be formulated as follows:


(6) \begin{equation*} {\mathrm{e}}_{ij}^{\phi }= at{t}_{node}\left({\mathrm{h}}_i^{\prime},{\mathrm{h}}_j^{\prime},\phi \right) \end{equation*}

where ${\mathrm{att}}_{node}$ refers to the deep neural network performing node-level attention. This network is shared for all node pairs based on the meta-path Φ, and their unique features determine the weight of each pair. It is important to note that while this process is asymmetric, meaning the importance of node *i* to node *j* and node *j* to node *i* can differ, node-level attention preserves this asymmetry. This is a crucial property of heterogeneous graphs.

Once having determined the importance of the meta-path-based node pairs, it will be normalized with the softmax function to obtain the weight coefficient ${\alpha}_{ij}^{\varnothing }$:


(7) \begin{equation*} {\alpha}_{ij}^{\phi }= soft\max \left({e}_{ij}^{\phi}\right)=\frac{\exp \left(\sigma \left({a}_{\phi}^T\cdot \left[{\mathrm{h}}_i^{\prime}\parallel{\mathrm{h}}_j^{\prime}\right]\right)\right)}{\sum_{k\in{N}_i^{\phi }}\exp \left(\sigma \left({a}_{\phi}^T\cdot \left[{\mathrm{h}}_i^{\prime}\parallel{\mathrm{h}}_k^{\prime}\right]\right)\right)} \end{equation*}

In this equation, σ represents the activation function, $\parallel$ represents the concatenate operation and ${\mathrm{a}}^{\mathrm{T}\Phi}$ is the node-level attention vector for meta-path Φ. The weight coefficient ${\alpha}_{ij}^{\varnothing }$ is asymmetric, meaning that it contributes differently to each other. Consequently, the meta-path-based embedding of node i can be gathered by the neighbor’s projected features, along with the corresponding coefficients, in the following manner:


(8) \begin{equation*} {z}_i^{\phi }=\sigma \left(\sum \limits_{j\in{N}_i^{\phi }}{\alpha}_{ij}^{\phi}\cdot{\mathrm{h}}_j^{\prime}\right) \end{equation*}

In this context, ${Z}_i^{\varnothing }$ refers to the learned embedding of node i for the meta path Φ. To address the above challenge, this article enhanced node-level attention to multi-head attention, making the training process more stable. The node level attention was repeated *K* times and combine the learned embeddings to create the semantic-specific embedding:


(9) \begin{equation*} {z}_i^{\phi }=\underset{k=1}{\overset{K}{\parallel }}\sigma \left(\sum \limits_{j\in{N}_i^{\phi }}{\alpha}_{ij}^{\phi}\cdot{\mathrm{h}}_j^{\prime}\right) \end{equation*}

After inputting node features into node-level attention using the meta-paths set {Φ_0_, Φ_1_, …, Φ_P_}, P distinct sets of semantic-specific node embeddings are generated. These sets are {Z_Φ0_, Z_Φ1_,., Z_ΦP_}.

### Semantic attention

The learned weights of each meta-path (β_Φ0_, β_Φ1_, …, β_ΦP_) can be displayed in the following manner:


(10) \begin{equation*} \left({\beta}_{\phi_0},{\beta}_{\phi_1},...{\beta}_{\phi_p}\right)= at{t}_{sem}\left({Z}_{\phi_0},{Z}_{\phi_1},...{Z}_{\phi_p}\right) \end{equation*}

We used ${\mathrm{att}}_{sem}$ to refer to a neural network carrying semantic-level attention. The importance of each meta-path is expressed as ${W}_{\varnothing i}$ as follows:


(11) \begin{equation*} {w}_{\phi_i}=\frac{1}{\left|V\right|}\sum \limits_{i\in V}{q}^T\cdot \tanh \left(W\cdot{z}_i^{\phi }+b\right) \end{equation*}

where *W* is the weight matrix, b is the bias vector and *q* is the semantic-level attention vector. Note that for the meaningful comparison, all the above parameters are shared for all meta-paths and semantic-specific embedding. After obtaining the importance of each meta-path, this article normalize them via the softmax function. The weight of meta-path ${\Phi}_{\mathrm{i}}$ denoted as ${\mathrm{\beta}}_{\Phi \mathrm{i}}$ can be obtained by normalizing the above importance of all meta-paths using the softmax function,


(12) \begin{equation*} {\beta}_{\phi_i}=\frac{\exp \left({w}_{\phi_i}\right)}{\sum_{i=1}^P\exp \left({w}_{\phi_i}\right)} \end{equation*}

It is important to note that meta-path ${\Phi}_{\mathrm{i}}$ becomes more significant as the value of ${\mathrm{\beta}}_{\Phi \mathrm{i}}$ increases. It is also worth mentioning that the weight of meta-path ${\Phi}_{\mathrm{i}}$ may vary depending on the task. Using the learned weights as coefficients, this article combine these semantic-specific embeddings and generate the final embedding *Z*:


(13) \begin{equation*} Z=\sum \limits_{i=1}^P{\beta}_{\phi_i}\cdot{Z}_{\phi_i} \end{equation*}

For *k*-order proximity, there are


(14) \begin{equation*} {Z}^{(k)}=\left\{\begin{array}{@{}l}X{\varTheta}^{(0)},k=0\\{}\frac{1}{2}\left({\varPi}^{(1)^{\frac{1}{2}}}{P}^{(1)}{\varPi}^{(1)^{-\frac{1}{2}}}+{\varPi}^{(1)^{-\frac{1}{2}}}{P}^{(1)^T}{\varPi}^{(1)^{\frac{1}{2}}}\right)X{\varTheta}^{(1)},k=1\\{}{W}^{(K)^{-\frac{1}{2}}}{P}^{(K)}{W}^{(K)^{\frac{1}{2}}} X\varTheta, k\ge 2\end{array}\right\} \end{equation*}

### Fusion

Finally, this article uses fusion operation Γ to fusion multi-scale features together as an Inception block ${Z}_I$:


(15) \begin{equation*} {Z}_I=\sigma \left(\varGamma \left({Z}^{(0)},{Z}^{(1)},...,{Z}^{(k)}\right)\right) \end{equation*}

This study aims to decrease the cross entropy of labeled nodes by improving the accuracy of predicted values in semi-supervised node classification:


(16) \begin{equation*} {L}_{T_{\mathrm{i}}}\left({f}_{\theta}\right)=-\left(\sum \limits_{l\in{y}_L}{Y}^l\log{f}_{\theta}\left({Z}_I^l\right)+\left(1-{Y}^l\right)\log \left(1-{f}_{\theta}\left({Z}_I^l\right)\right)\right) \end{equation*}

The parameter θ defines the Meta-DHGNN model. It includes ${y}_L$, a collection of labeled node indexes and ${Y}^I$ and ${Z}^I$, which represent the label and embedding information for the nodes. *T*_i_ refers to a batch of samples from the meta-training task, denoted as *T_i_* ~ *P*(*T*), where *P*(*T*) indicates the distribution of the meta-training task distributed across the training set.

### Statistics analysis

In this study, we employ various statistical analysis formulas, including F1 score, recall rate and precision rate. These three formulas have been utilized for analyzing the ACM, IMDB and cytokines datasets. The F1 score represents a harmonic mean of accuracy and recall rates. The magnitude of the F1 score reflects the model’s performance, while recall and precision rates provide detailed insights.


(17) \begin{equation*} Precision= TP/\left( TP+ FP\right) \end{equation*}

(18) \begin{equation*} Recall= TP/\left( TP+ FN\right) \end{equation*}

(19) \begin{equation*} F1={2}^{\ast }\ \left({Precision}^{\ast }\ Recall\right)/\left( Precision+ Recall\right) \end{equation*}

### Experiments and results

#### Verify model performance

The performance of the model is individually validated in this study using two disassembly methods: (1) incorporating meta-learning into the heterogeneous model leads to noticeable changes in its performance, particularly for small sample datasets. (2) Through targeted modifications, heterogeneous models combine the advantages of directed modules on different datasets.

For each method, experiments were conducted to verify their impact on both publicly available and biological datasets developed within this study. Furthermore, experimental validation and previous research findings demonstrate that the model employed in this study outperforms similar models.

#### Experimental details and performance comparison

The experiment in this study utilizes the Adam optimization algorithm. Due to the incorporation of meta-learning in our model, it exhibits a relatively high number of hyperparameters, thereby necessitating avoidance of grid search for parameter optimization. Instead, we adopted a method of individually adjusting one parameter while keeping the others fixed. We conducted experiments by testing 10 values within a specific range for each parameter and evaluated their impact on the model’s performance. The value corresponding to the best model performance was selected as the optimal setting for that particular hyperparameter. This process was repeated for other hyperparameters as well. Due to the abundance of graphic files, we have included them as attachments in the paper for convenient access and reference (Figures S1–S3). We exclusively present the parameter representation of the Meta-DHGNN model in this section, while the parameters of other models can be found in the supplementary materials. Regarding data sets from ACM, the learning rate for the meta-learning component is set to 0.01, and the L2 regularization parameter is set to 0.001. For iterative training, the maximum number of epochs is limited to 50, with two meta-graphs selected and 30 instances from each category used for training at a time for training purposes, while dropout is set to 0.7. After meta-learning, the learning rate is adjusted to 0.008, the L2 regularization parameter remains at 0.001 and the number of epochs increases to 150. The meta-learning model achieves its peak performance at epoch 40, followed by the utilization of this metamodel for formal training. Subsequently, the formally trained model attains optimal performance at epoch 52. The learning rate of the meta-learning component is set to 0.003 and the L2 regularization parameter is set to 0.002 for data sets obtained from IMDB. During iterative training, a maximum of 50 epochs are allowed, with two meta-graphs and 30 instances selected from each class at a time for training purposes, while dropout is set to 0.9. Following meta-learning, the learning rate is adjusted to 0.008, maintaining the L2 regularization parameter at 0.006 and increasing the number of epochs to 150. The metamodel achieves its peak performance at epoch 18 before being formally trained using it as a basis. Subsequently, the formally trained model reaches its peak performance at epoch 77.

The dimension of semantic level attention vector q is set as 128 units. Additionally, the *K* value is chosen as 8. Simultaneously, an early stop threshold is defined while preserving most existing model parameters and evaluation metrics to mitigate ineffective training. The obtained test results are presented in Table 2.

**Table 2:** Experimental results. The performance results of our Meta-DHGNN and the heterogeneous model on the ACM and IMDB datasets, respectively, demonstrate that our model outperforms the heterogeneous model

Model	ACM		
	Macro precision	Macro recall	Macro f1
dglhan	0.8912	0.8824	0.8842
Meta-DHGNN	0.8994	0.8955	0.8964
	**IMDB**		
	**Macro precision**	**Macro recall**	**Macro f1**
dglhan	0.5652	0.5568	0.5533
Meta-DHGNN	0.5712	0.5649	0.5664

**Figure 3:**
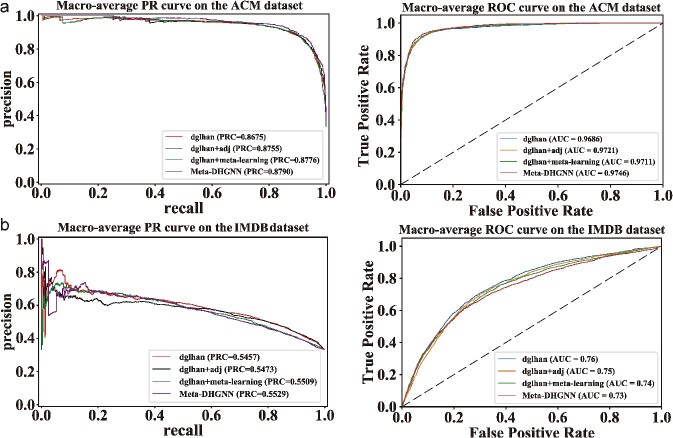
Experimental PR and AUC curves. A indicates the PR curve and ROC curve of the ACM data set. B represents PR curve and ROC curve of IMDB data set. Them represents PR and AUC curves of two datasets under different improvements to see the effects of different models on different datasets in more detail.

This study adopted a multi-perspective approach to examine the model, aiming for more specific and nuanced results. Specifically, our experiment focused on comparing the performance of the model in its directed and meta-learning modules. The experimental results for two datasets under various modes are presented in Table 3. However, meta-learning remained unaffected by these changes. Furthermore, it is worth mentioning that the impact of meta-learning was marginally superior to that of directed change. *K*-order approximation in a digraph convolution can be achieved larger receptive fields and learn multi-scale features in graphs. Therefore, even in heterogeneous data sets, its effect will be somewhat improved. Additionally, this study conducted an analysis of AUC and PRC measures for each case. The analysis results are presented in Figure 3, where a and represent the outcomes of PRC and AUC. This study shows that Meta-DHGNN outperforms heterogeneous networks in terms of performance and shows competitive results.

**Table 3:** Detailed experimental results. In the ablation experiment, we can observe the impact of each module on model performance, and incorporating meta-learning and directed modules significantly enhances model performance

Model	ACM		
	Macro precision	Macro recall	Macro f1
dglhan	0.8912	0.8824	0.8842
dglhan+adj	0.8968	0.8868	0.8866
dglhan+meta-learning	0.8992	0.8908	0.8919
Meta-DHGNN	0.8994	0.8955	0.8964
	**IMDB**		
	**Macro precision**	**Macro recall**	**Macro f1**
dglhan	0.5652	0.5568	0.5533
dglhan+adj	0.5661	0.5584	0.5606
dglhan+meta-learning	0.5650	0.5641	0.5634
Meta-DHGNN	0.5712	0.5649	0.5664

The experimental results indicate that the proposed method has demonstrated favorable performance on ACM and IMDB datasets. The inclusion of a small sample learning mechanism in the study model aligns better with the limited training data available. Moreover, utilizing directed relationships provides a more comprehensive representation of network connections. In comparison to other graph optimization algorithms such as the graph convolutional neural network (GCN) and graph attention network (GAT), which have shown promising outcomes in heterogeneous networks [7]. Table 4 presents the obtained results for the two datasets.

**Table 4:** Performance comparison chart. The training results of two datasets in GCN and GAT models are easy to compare with the model in this study

Model	ACM (Macro f1)	IMDB (Macro f1)
GCN	0.8681	0.4573
GAT	0.8623	0.4944
Meta-DHGNN	0.8964	0.5664

### Properties of Meta-DHGNN on cytokines and proteins

In this experiment, the prediction task of related cytokines can be considered as a secondary classification problem within the broader context of classifying whether current cytokines are associated with CRS. In the above experiments, the advanced nature of the Meta-DHGNN model in performing classification tasks has been effectively proved. On that basis, this study incorporates a dataset comprising 1615 cytokines and proteins. The prediction results exhibit varying probabilities of being linked to CRS [22]. To facilitate the model’s assessment on the biological dataset, a total of 200 cytokines and proteins were selected from both ends of the Meta-GNN probabilistic sequencing results to predict CRS-related cytokines for classification testing.

**Figure 4:**
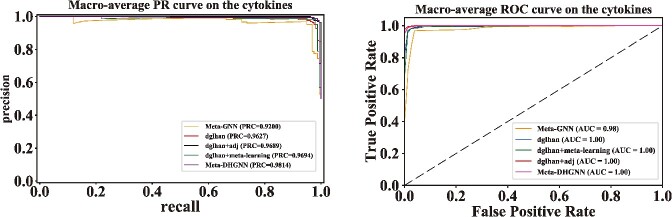
AUC and PR curves were generated to evaluate the performance of different models in predicting cytokine datasets. The ROC-AUC curve and PR curve were displayed for comparison. It is evident that the overall effect has been significantly improved.

We exclusively present the parameter representation of the Meta-DHGNN model in this section, while the parameters of other models can be found in the supplementary materials. The learning rate for the meta-learning component in the experiment is set to 0.001, while the regularization parameter is set to 0.001. In terms of iterative training, a maximum of 50 epochs are allowed, with two meta-graphs selected and trained using a batch size of 30 samples per category. The dropout is set to 0.9. The learning rate is set to 0.001 after meta-learning, while the regularization parameter is set to 0.001 and the number of epochs is set to 150. The dimension of the semantic level attention vector q is specified as 128, with a total of eight attention heads (*K*). The metamodel achieves its peak performance at epoch 25 before being formally trained using it as a basis. Subsequently, the formally trained model reaches its peak performance at epoch 22. Simultaneously, an early stop threshold is defined along with preserving most existing model and evaluation indicators in order to mitigate ineffective training. The experimental results are presented in Table 5, while Figure 4 illustrates the loss curve.

**Table 5:** Testing of cytokine data sets. In the cytokine dataset, our proposed Meta-DHGNN model outperforms the Meta-GNN model from previous studies in terms of performance. Furthermore, through ablation experiments, we observe the impact of each module’s incorporation on enhancing the model’s performance

Model	Macro precision	Macro recall	Macro f1
Meta-GNN	0.9504	0.9481	0.9487
dglhan	0.9684	0.9688	0.9687
dglhan+adj	0.9753	0.9750	0.9750
dglhan+meta-learning	0.9812	0.9812	0.9812
Meta-DHGNN	0.9875	0.9875	0.9875

### Biological discovery

Meta-DHGNN was used to investigate the CRS-associated cytokines in CAR-T therapy upon the constructed directed heterogeneous cytokine interaction network.

In the experiment, the prediction task can be regarded as a binary classification problem: determining whether the current cytokine is related to CRS. In detail, model training was carried out with Meta-DHGNN, and all network nodes were subsequently predicted about CRS with the trained model.

Before training, a literature mining study confirmed 23 cytokines or cells related to CRS as the positive nodes. These included IL1A, IL1B, IL2, IL2RA, IL4, IL5, IL6, IL10, IL13, IL15, IL17A, TNF, CCL2/MCP-1, CCL3/MIP-1A, CCL4/MIP-1B, CSF2/GM-CSF, CXCL10/IP-10, CXCL8/IL-8, CXCL9/MIG, IFNG, T cells, endothelial cells and macrophages. Additionally, 30 nodes with the farthest distance from the positive nodes were initially chosen as the negative.

**Figure 5:**
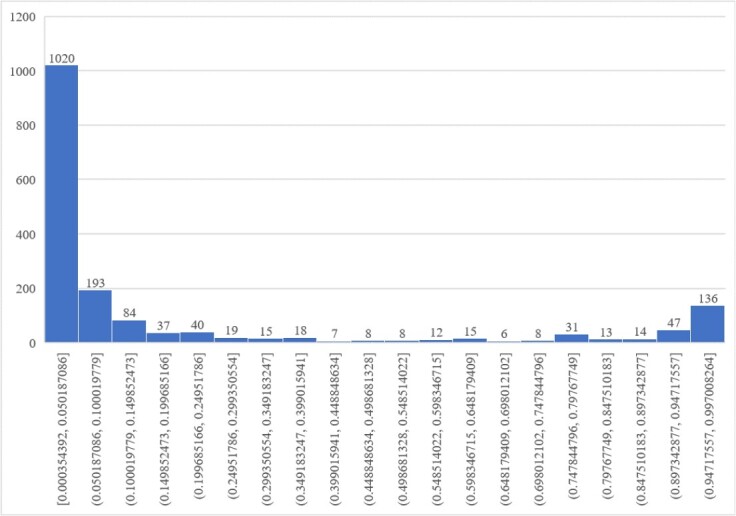
The probability distribution histogram of the predicted result. The results revealed two peaks, one on the left and another on the right, with the latter being lower due to a higher proportion of negative labels compared to positive ones. Consequently, selecting 136 cytokines from the far-right region based on these findings would effectively minimize false positives.

Adam was used to optimize the model parameters. The learning rate was set to 0.001 in the meta-learning phase, and the L2 regularization weight was 0.001. The maximum number of epochs was 50. The number of meta-learning tasks was set to 2, with 35 of each category for training. After the meta-learning, the learning rate was set to 0.001, and the L2 regularization weight was 0.001. The maximum number of epochs was 150. The metamodel achieves its peak performance at epoch 43 before being formally trained using it as a basis. Subsequently, the formally trained model reaches its peak performance at epoch 24. The semantic level attention vector q dimension was set to 128, and the number K was 8. The dropout rate was set to 0.9. Meanwhile, the early stop was adopted to reduce invalid training. In order to prevent the initial data selection from affecting the prediction outcome, the process was repeated many times. The median prediction value for each cytokine was then selected as the final prediction result, as illustrated in Figure 5.

The cytokine prediction results of each data segment, as depicted in Figure 5, exhibit a consistent pattern of high values on both ends and low values in the middle. This observation signifies the model’s efficacy in data classification. We selected 136 data points from the far right as our final prediction outcome.

These specific data points, along with their corresponding probability values (as presented in Table 6), strongly indicate a significant association between these cytokines and positive labels, thereby predicting a high likelihood of CRS association in CAR-T therapy.

**Table 6:** The probability of cytokines predicted by the Meta-DHGNN. The higher the probability, the closer the relationship with CRS

Cytokines	Probability	Cytokines	Probability	Cytokines	Probability	Cytokines	Probability
IL1B	0.997027	IL23A	0.995815	HLA-DPB1	0.990785	CCL28	0.978556
IL4	0.99702	CCL20	0.995606	CCR5	0.990773	ZAP70	0.978485
IL12B	0.997019	IL2RA	0.995605	IL23R	0.989595	IL6ST	0.977602
IFNG	0.997008	CCL11	0.995601	STAT5B	0.989472	IL10RA	0.977456
IL12A	0.997008	IFNGR1	0.995576	RELA	0.989391	IL10RB	0.977228
TNF	0.997002	HLA-DRA	0.995508	IL9	0.989221	CCL7	0.977124
CXCL8	0.996999	IFNGR2	0.995486	STAT6	0.989097	CCL21	0.976976
IL10	0.996992	IL1R1	0.99548	STAT1	0.988952	STAT5A	0.976824
IL5	0.996986	CCL3L3	0.995371	CD40LG	0.988741	IL1R2	0.971843
IL6	0.996977	IL18R1	0.994965	IL5RA	0.988528	IL1RN	0.971433
CCL5	0.996973	FOS	0.994948	CCL22	0.988342	CASP1	0.970305
IL2	0.996972	IL4R	0.994858	CXCL13	0.987989	LCK	0.969954
IL13	0.996972	JAK1	0.994692	MAPK9	0.987975	TGFB1	0.968218
IL1A	0.996972	TAB3	0.99453	CD40	0.987968	FAS	0.968072
CSF2	0.996952	CCL19	0.994483	NFATC1	0.987896	SQSTM1	0.967794
CCL3	0.996929	IL12RB1	0.994398	CD4	0.987465	CTLA4	0.9655
CCL4	0.996926	CXCL5	0.994199	IL18RAP	0.987046	MAPK8	0.965382
CXCL10	0.99692	CXCL6	0.993855	IRAK4	0.987038	IFNA1	0.964629
IL17A	0.99684	CD28	0.993713	CCR1	0.986899	IFNA2	0.964567
CCL2	0.996777	PTGS2	0.993492	IL6R	0.986643	IFNAR2	0.963792
IL18	0.996746	IL17F	0.993221	CCR2	0.986542	TLR5	0.963164
CXCL9	0.996711	IL21	0.993218	MAP3K7	0.986349	CXCR5	0.963067
IL2RG	0.996604	IFNA13	0.992903	MYD88	0.986067	CCL25	0.962745
TNFRSF1A	0.996519	TBK1	0.992488	JUN	0.985923	CX3CL1	0.9608
CXCL11	0.996504	MAP3K8	0.992223	TRAF6	0.985717	IL25	0.960522
IL2RB	0.99642	IL12RB2	0.991862	IRAK1	0.985459	MAPK14	0.959187
NFKBIA	0.996417	IKBKB	0.991837	CHUK	0.9852	CSF2RB	0.958946
NFKB1	0.996389	JAK3	0.991773	IL21R	0.984914	IL17RC	0.955399
CD80	0.996309	IL22	0.991603	MAP2K4	0.981676	IL37	0.955147
IL15	0.996297	STAT4	0.991555	STAT3	0.981425	IRAK2	0.953217
CASP8	0.99619	TAB2	0.991477	MAP2K7	0.981109	IFNAR1	0.952774
CD86	0.996059	IL3	0.991042	CCL1	0.980998	TLR4	0.950902
CXCL1	0.996048	FADD	0.990933	CCL17	0.980387	IL17D	0.95084
IFNB1	0.995934	CXCL12	0.990812	IKBKG	0.980134	TBX21	0.950808

**Figure 6:**
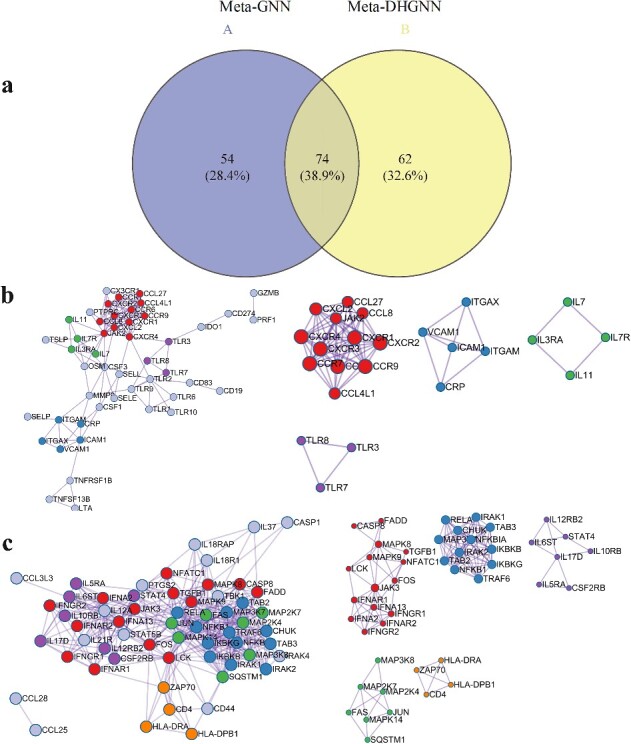
Protein interaction network of associated cytokines. (**A**) Comparison of the two models. (**B**) Predicted by Meta-GNN model. (**C**) Predicted by Meta-DHGNN model.

The comparison between the predictions of the Meta-DHGNN and Meta-GNN [22] is shown in Figure 6, showing that 74 cytokines are in common and 62 are unique upon a more accurate cytokine interaction network.

For both studies, interaction networks were created only between associated cytokines. Meta-GNN predicted a network that can be divided into four modules, with the first focusing on chemokines. Chemokines are cytokines that assist in recruiting and transporting leukocytes for immune response. The remaining three modules consist of inflammatory markers in the blood, members of the Toll-like receptor family and additional cytokines. In this study, Meta-DHGNN predicted a network that can be divided into five modules, with the first module centered on IFN receptor proteins and cytokines in the MAPK pathway. The remaining four modules mainly comprise the follow-up cytokines in the pathway. This suggests that biological processes are more closely related to pathways.

In detail, the predicted IFN family and its receptors include IFNG (IFN-γ), IFNA1, IFNB1, IFNA13, IFNA2, IFNAR1, IFNAR2, IFNGR1 and IFNGR2. IFN-γ with TNF-α and IL-6 are markers of COVID-19 cytokine storm [23] and are highly involved in various cytokine storm–related diseases [24]. Besides, the severity of the COVID-19 cytokine storm and its relationship to IFNA and IFNB has been demonstrated in some studies [25]. Moreover, activating the Toll-like receptor pathway will also promote the cascade reaction of MAPK and IFN [25–27], further aggravating inflammatory reaction, making them play an indispensable role in the occurrence and progress of CRS.

## DISCUSSION

This study’s prediction results, using the new topological relationship network, are more concentrated on IFN and its family factors than previous studies. This suggests that these factors have a crucial role in the CRS caused by CAR-T therapy.

**Figure 7:**
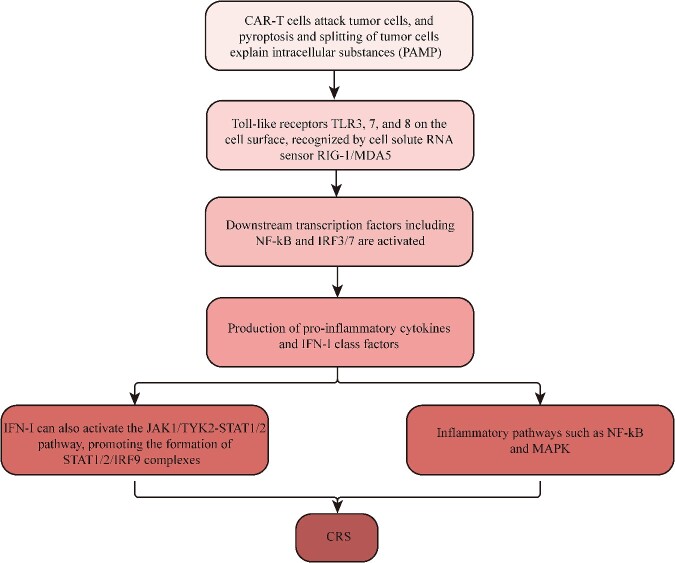
The pathway mechanism of CRS. The diagram illustrates the mechanistic pathway of CRS induced by CAR T cell therapy. Upon tumor cell recognition and subsequent attack by CAR T cells, intracellular substances are released from lysed tumor cells, ultimately culminating in the development of CRS. The depicted figure demonstrates the interplay between these key components within this pathway.

Regarding the mechanism by which CRS occurs in CAR-T therapy, as illustrated in Figure 7 when CAR-T cells attack tumor cells, the tumor cells experience pyroptosis and lysis, releasing intracellular substances known as pathogen-related molecular models (PAMPs). In endosomes, TLR3, 7 and 8 Toll-like receptors and the RIG-1/MDA5 cytosolic RNA sensor are responsible for detecting viruses. IFN-I can activate inflammatory pathways such as NF-kB and MAPK, leading to the overproduction of proinflammatory cytokines, causing an excessive inflammatory response (CRS) in COVID-19 [28]. IFN-I can also activate the production of proinflammatory cytokines and IFN-I simultaneously by activating two downstream transcription factors, NF-kB and IRF3/. Additionally, IFN-I can activate the JAK1/TYK2-STAT1/2 pathway, which promotes the formation of the STAT1/2/IRF9 complex.

**Figure 8:**
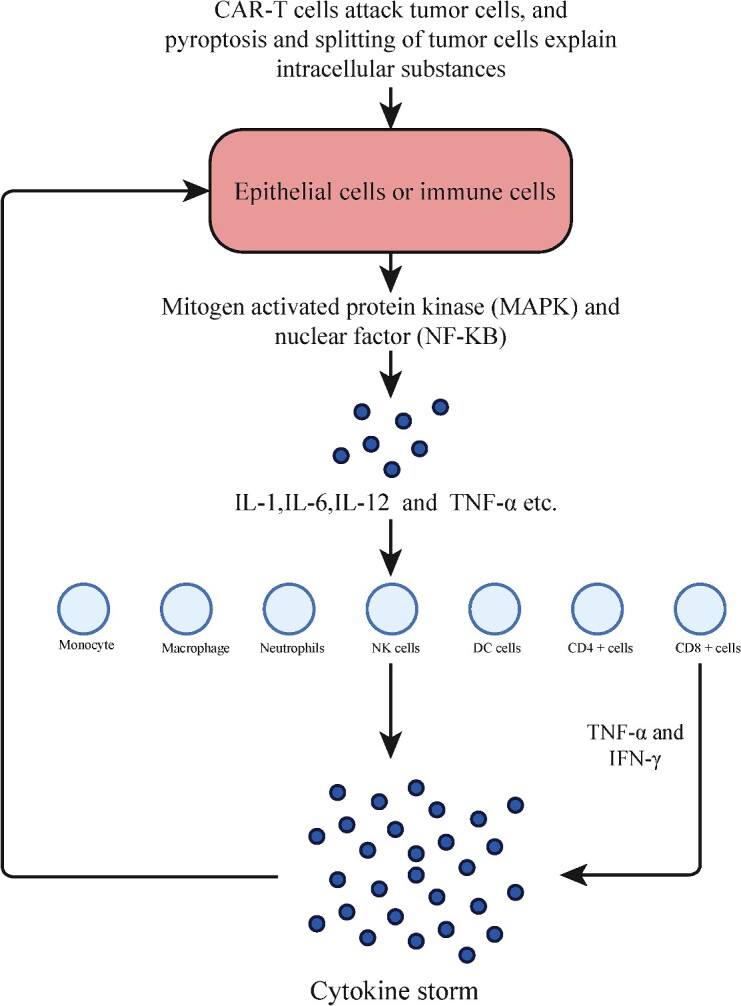
Immune mechanism of CRS. This figure depicts the immunological mechanism underlying CRS induced by CAR-T cell therapy. Following recognition and subsequent attack of tumor cells by CAR T cells, intracellular substances are released from lysed tumor cells, triggering cellular responses and release of key cytokines that ultimately contribute to CRS development. The diagram illustrates the immunological interplay between these cells and their associated cytokines.

In Figure 8, it is shown that PAMP can trigger immune or epithelial cells, leading to tissue damage. This can release inflammatory cytokines, such as IL-1, IL-6, IL-12 and TNF-α. These cytokines can activate macrophages to produce IL-6 by activating mitogen-activated protein kinase MAPK and nuclear factor NF-kB. These inflammatory cytokines can also recruit innate immune cells (monocytes, macrophages, neutrophils, DC and NK cells) and activate adaptive immune cells (CD4 + cells and CD8 + cells). This can lead to bone marrow cytogenesis and acute granulopoiesis, as well as the production of excessive circulating cytokines. This can further exacerbate epithelial damage and trigger a cytokine storm [28], causing CRS. It is important to note that these cytokine storms can cause damage to the human body.

**Figure 9:**
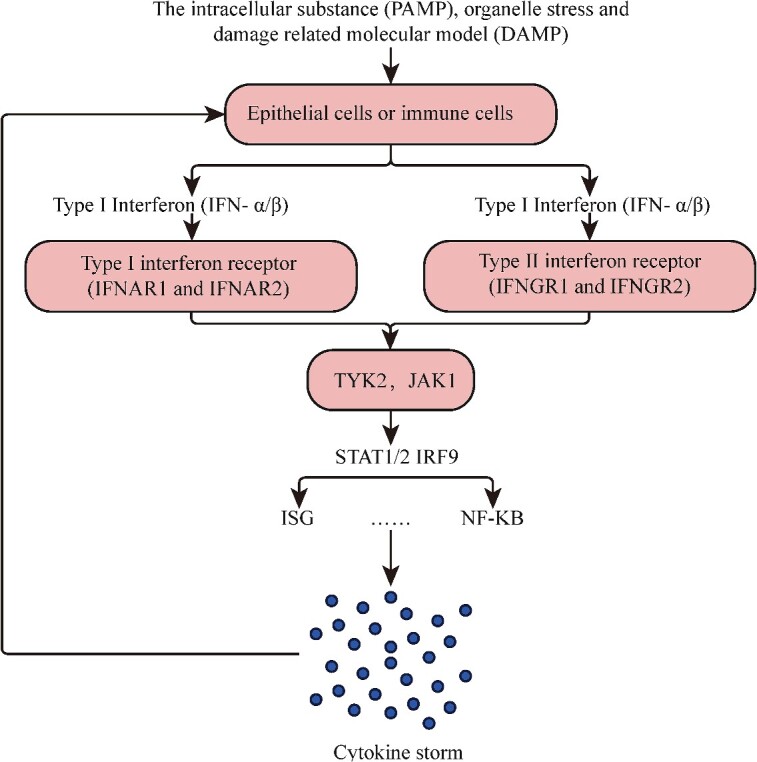
The role of the IFN family in CRS. This diagram illustrates the mechanism underlying CRS induced by chimeric antigen receptor T-cell (CAR-T cell) treatment, focusing on the involvement of IFN family members and their receptors in mediating CRS development following CAR-T cell recognition and subsequent attack on tumor cells.

In this study, we explored the mechanism of IFN-related cytokines in the development of CRS. The development of CRS was investigated in this study, focusing on the mechanism of IFN-related cytokines. Interferon is a type II cytokine that is induced by pathogen-related molecular patterns and stress stimuli, such as DNA damage, organelle stress and damage-related molecular patterns (DAMPs), as seen in Figure 9. Human type I interferon (IFN-α/β) signals through heterodimer type I interferon receptors (IFNAR1 and IFNAR2), while IFN-γ is the only acidic, unstable type II interferon that binds to type II interferon receptors (IFNGR1 and IFNGR2) [29]. IFN-α/β has a broad cross-cell- type synthesis after participating in pattern recognition receptor PRR and activation of transcription factor TF (including IFN regulatory factors IRF and NF-kB) [30]. The downstream signal transduction of IFN-α/β depends on JAK (JAK1, TYK2, JAK2) phosphorylation of STAT1 and STAT2. TYK2 is necessary for stimulation in response to IFN-α/β [31], as it acts as a scaffold for IFNAR1 and prevents premature degradation [32]. The activated STAT binds to IRF9 to form a heterotrimeric ISGF3 transcriptional complex, which is necessary to transduce IFN-stimulated response-dependent ISG [29]. IFN induces the IFN-stimulating gene (ISG), which controls cell proliferation and metabolism and exerts pleiotropy. Immune cells can also activate NF-kB and MAPK pathways through unconventional means, releasing proinflammatory cytokines and excessive inflammation, in addition to their role in antigen presentation, cell recruitment and activation. Medical professionals and researchers can benefit from discovering the Meta-DHGNN model in predicting, preventing and controlling CRS.

### Limitation

Despite achieving promising results in predicting potential cytokines and some datasets, Meta-DHGNN still has certain limitations:

(i) The parameters of Meta-DHGNN are multiple; a more satisfactory approach can be employed to obtain the appropriate parameters. Additionally, the functional similarity of directed heterogeneous networks relies on the richness and information accuracy of known directed heterogeneous networks. As experimental verification continues to uncover more interactions in biological networks, the predictive performance of this model for such networks will be further enhanced.
(ii) The Meta-DHGNN algorithm, based on deep learning, exhibits adaptability to oriented and heterogeneous network learning. However, the challenge of obtaining enhanced embeddings and identifying more precise features in such networks remains an area that requires further exploration.
(iii) The progression of human diseases is a multifaceted process, and in the foreseeable future, numerous analogous biological-directed and heterogeneous network associations will be unveiled. Investigating the interplay among cytokines or other proteins will aid in elucidating the underlying pathogenic mechanisms.

## CONCLUSION

In this study, we propose Meta-DHGNN, a meta-learning-based analysis method for directed and heterogeneous GNNs. The integration of both directed and heterogeneous algorithms in our proposed method addresses the challenge of limited data availability effectively. This approach enables comprehensive analysis of the cytokine network and accurate prediction of CRS-related cytokines.

Firstly, to overcome the limitation posed by small datasets, we conduct a pre-training phase using the meta-learning module. Consequently, the directed algorithm constructs an adjacency matrix that captures potential relationships more realistically. Ultimately, the heterogeneous algorithm utilizes meta-photographs and multi-head attention mechanisms to enhance the verisimilitude and precision of cytokine information prediction associated with positive labels. Our experimental validation on the dataset demonstrates that Meta-DHGNN achieves favorable outcomes. Moreover, based on the predicted results, we have explored the multifaceted etiology of CRS in CAR-T therapy from diverse perspectives and identified several overlooked cytokines, such as IFNG (IFN-γ), IFNA1, IFNB1, IFNA13, IFNA2, IFNAR1, IFNAR2, IFNGR1 and IFNGR2 that potentially play pivotal roles.

The significance of Meta-DHGNN lies in its capacity to effectively analyze directed and heterogeneous networks in biology, while also facilitating the prediction of CRS risk in CAR-T therapy. This can facilitate physicians and drug developers in identifying potentially significant indicators, enabling them to provide corresponding clinical and drug design guidance. Additionally, it aids researchers in discovering a more comprehensive array of crucial cytokines associated with CRS.

**Key Points**

- At present, many algorithms cannot deal with directed and heterogeneous relationships in biological data processing. However, many real relationships in biological data are directed and heterogeneous. For this purpose, we propose a meta-learning-based directed heterogeneous network, Meta-DHGNN, to predict cytokines, and our model can be extended to other aspects for prediction.
- Based on a large number of literatures, we construct a relatively rich oriented and heterogeneous cytokine topology.
- By effectively extracting salient features and accurately discerning relationships among directed and heterogeneous data, our model exhibits robust performance across diverse datasets, encompassing ACM-, IMDB- and CRS-associated cytokine datasets.
- We predicted 136 cytokines related to CRS based our model prediction. We found the new IFN family are important to explain the origin of CRS, whose importance are not shown strongly in the prediction of previous study.

## Supplementary Material

supplementary_bbae104

## Data Availability

Our program is in https://github.com/wzy38828201/Meta-DHGNN. All data in our study are available upon request (please contact the corresponding authors).

## Notes

**Zhenyu Wei** is the doctoral student at Harbin Engineering University.

**Chengkui Zhao** is the director of AI center of Shanghai Unicar-Therapy Bio-medicine Technology Co., Ltd.

**Min Zhang** is a doctoral student at Harbin Engineering University.

**Jiayu Xu** is the doctoral student at Harbin Engineering University.

**Nan Xu** is the R&D Director of Shanghai Unicar-Therapy Biomedicine Technology Co., Ltd.

**Shiwei Wu** is the doctoral student at Harbin Engineering University.

**Xiaohui Xin** is the master student at Harbin Engineering University.

**Lei Yu** is the CEO of Unicar-Therapy Bio-medicine Technology Co., Ltd.

**Weixing Feng** is the professor at Harbin Engineering University.
