# Supplementary material for: Meta-DHGNN: method for CRS-related cytokines analysis in CAR-T therapy based on meta-learning directed heterogeneous graph neural network
Source: Brief Bioinform. 2024 Mar 27;25(3):bbae104. doi: 10.1093/bib/bbae104 (PMC10976917; doi:10.1093/bib/bbae104)
Supplement: supplementary_bbae104 [file supplementary_bbae104.docx]

**Figure S1**. The performance graph illustrates the model tuning results on the ACM data set, where a, b, c, d, e, f, and g represent variations in performance with fixed remaining hyperparameters solely for the horizontal axis hyperparameter. Among these variations, a, b and c are shared hyperparameters across all four models while d, e, f and g are unique to the meta-learning model. Although the order in which the model hyperparameters are fixed varies slightly from model to model, the data in the final figure indicates the optimal result achieved by the model.





**Figure S2**. The performance graph illustrates the model tuning results on the IMDB data set, where a, b, c, d, e, f, and g represent variations in performance with fixed remaining hyperparameters solely for the horizontal axis hyperparameter. Among these variations, a, b and c are shared hyperparameters across all four models while d, e, f and g are unique to the meta-learning model. Although the order in which the model hyperparameters are fixed varies slightly from model to model, the data in the final figure indicates the optimal result achieved by the model.





**Figure S3**. The performance graph illustrates the model tuning results on the Cytokines data set, where a, b, c, d, e, f, and g represent variations in performance with fixed remaining hyperparameters solely for the horizontal axis hyperparameter. Among these variations, a, b and c are shared hyperparameters across all four models while d, e, f and g are unique to the meta-learning model. Although the order in which the model hyperparameters are fixed varies slightly from model to model, the data in the final figure indicates the optimal result achieved by the model.
